# Automated inter-rater reliability assessment and electronic data collection in a multi-center breast cancer study

**DOI:** 10.1186/1471-2288-7-23

**Published:** 2007-06-18

**Authors:** Soe Soe Thwin, Kerri M Clough-Gorr, Maribet C McCarty, Timothy L Lash, Sharon H Alford, Diana SM Buist, Shelley M Enger, Terry S Field, Floyd Frost, Feifei Wei, Rebecca A Silliman

**Affiliations:** 1 Geriatrics Section, Department of Medicine, Boston University School of Medicine, Boston, Massachusetts, USA; 2 Department of Biostatistics, Boston University School of Public Health, Boston, Massachusetts, USA; 3 Department of Epidemiology, Boston University School of Public Health, Boston, Massachusetts, USA; 4 HealthPartners Research Foundation, Minneapolis, Minnesota, USA; 5 Henry Ford Health System, Detroit, Michigan, USA; 6 Center for Health Studies, Group Health, Seattle, Washington, USA; 7 Department of Research and Evaluation, Kaiser Permanente Medical Care Program, Pasadena, California, USA; 8 Meyers Primary Care Institute of Fallon Community Health Plan/Fallon Foundation/University of Massachusetts Medical School, Worcester, Massachusetts, USA; 9 Lovelace Respiratory Research Institute, Albuquerque, New Mexico, USA

## Abstract

**Background:**

The choice between paper data collection methods and electronic data collection (EDC) methods has become a key question for clinical researchers. There remains a need to examine potential benefits, efficiencies, and innovations associated with an EDC system in a multi-center medical record review study.

**Methods:**

A computer-based automated menu-driven system with 658 data fields was developed for a cohort study of women aged 65 years or older, diagnosed with invasive histologically confirmed primary breast cancer (N = 1859), at 6 Cancer Research Network sites. Medical record review with direct data entry into the EDC system was implemented. An inter-rater and intra-rater reliability (IRR) system was developed using a modified version of the EDC.

**Results:**

Automation of EDC accelerated the flow of study information and resulted in an efficient data collection process. Data collection time was reduced by approximately four months compared to the project schedule and funded time available for manuscript preparation increased by 12 months. In addition, an innovative modified version of the EDC permitted an automated evaluation of inter-rater and intra-rater reliability across six data collection sites.

**Conclusion:**

Automated EDC is a powerful tool for research efficiency and innovation, especially when multiple data collection sites are involved.

## Background

Advances in computer technology have produced readily available, low cost, portable, highly efficient computer equipment. Coupled with the transition of medical documentation from paper to electronic storage, computer technology has helped to facilitate changes in data collection for clinical research. The choice between paper data collection methods or automated electronic data collection (EDC) methods has become a key design question for clinical researchers. However, the literature on electronic data collection is sparse. We identified only a few published reports that have compared EDC with paper data collection, or more generally addressed EDC for research purposes. [1-6]

Researchers have turned to electronic methods of data collection to improve the quality of data and to conduct research more effectively and efficiently. Standardizing the data collection process to maintain cross-site data consistency remains a challenge in multi-center studies. Given the required investment in hardware, software, and training, researchers must consider the advantages and disadvantages of adopting EDC.[7] Some of the documented advantages of EDC are: (1) integration of mixed data types by preloading electronically available data and manually inputting non-electronic data; (2) programmed error checking by transparent decision algorithms; (3) directed data entry by use of pick lists, forced data entry, and automated skip patterns; (4) automated validation procedures such as data reliability and data range checks; (5) increased opportunity for innovation; and (6) reduced time from study implementation to manuscript submission. [1-8] The disadvantages are: (1) extensive time and programming needed to develop EDC systems; (2) equipment costs; and (3) lack of ability to verify miscoded data against paper records, once data have been entered. [1-8] Despite these disadvantages, EDC offers promise for integrating existing data for multi-site longitudinal studies with flexibility, innovation, and less effort than that required by traditional paper methods. In addition, subsequent systems can be developed from the first system more efficiently and at lower cost.[8]

We report on our experience with an EDC system designed to collect data for a multi center breast cancer study, and an innovative method to assess cross-site data consistency with a subsequent system automating inter-rater/intra-rater reliability strategy.

## Methods

Cohort ascertainment and data collection processes have been described elsewhere.[9] In brief, we identified potentially eligible subjects for inclusion in a cohort study from electronic databases at six of the Cancer Research Network (CRN) health care delivery systems: Group Health, Washington; Kaiser Permanente Southern California; Lovelace, New Mexico; Henry Ford Health System, Michigan; Health Partners, Minnesota; and Fallon Community Health Plan, Massachusetts. The CRN is a consortium of healthcare delivery systems, funded by the National Cancer Institute to increase the effectiveness of preventive, curative and supportive interventions that span the natural history of major cancers.

Women aged 65 years or older and diagnosed with primary, histologically confirmed, early stage unilateral breast cancer between January 1, 1990 and December 31, 1994 were eligible for inclusion in the study. Before the start of medical record-based data collection, we collected electronically demographic, tumor, treatment, and surgeon data from cancer registry, administrative, and clinical databases at each site. Medical record abstractors verified (at sites with cancer registries) or determined (at sites without cancer registries) eligibility for each identified subject and collected any information that had not been available from electronic sources. We abstracted data from the date of initial diagnosis through death, disenrollment from the health plan or 10 years post-diagnosis. There were no control or intervention strategies in this study. We describe the variability in electronically available data across six sites and report efficiencies gained from implementing an electronic data collection (EDC) instead of the originally proposed paper-based data collection.

### The EDC system

**Figure 1:**
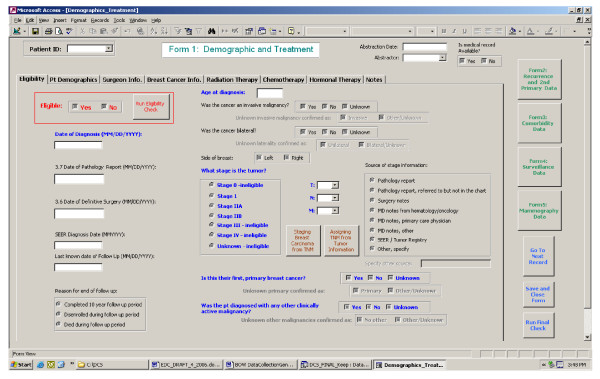
Screen shot of EDC data collection form.

We developed a computer-based automated menu-driven EDC system using Microsoft^® ^Access 2000. The "back end" of the system consisted of six tables that stored 658 exported data variables after completion of data abstraction in the "front end" of the system. The "front end" of the system was organized into five forms for collecting information on (1) eligibility, patient characteristics, tumor characteristics, and treatment, (2) diagnosis and treatment of recurrence and second primary cancer, (3) comorbidity at three time points, (4) surveillance testing for recurrent breast cancer after completing primary therapy, and (5) mammography screening. All forms were menu-driven, in a tabular format (as shown in Figure 1), and linked by a unique study subject identification (ID) number. Each site maintained a Microsoft^® ^Excel file that linked each subject's study number in the EDC to the subject's original medical record number to allow local access to electronic data and medical records as needed. Study specific queries and macros were programmed to allow for toggling between the forms, verification of input data, final checking for completeness of the data collected, and for export into the "back-end" database. Consistent with data use agreements between Boston Medical Center and the data collection sites, and per HIPAA agreements, personal identifiers such as surgeons' names and patients' day of birth were deleted before exporting into the "back-end" database.

For each site, we preloaded into the EDC all study ID numbers for potentially eligible subjects along with all electronically available data. Each site-specific EDC system was configured for use on individual computers. To ensure cross-site consistency, one person trained medical record abstractors at each participating site using local, de-identified medical records as sample records. For enhanced efficiency a 10% sampling scheme was implemented to capture a sub-sample of the stage I, non-Hispanic White, less than 80 years age group at Kaiser Permanente Southern California, the largest site.

To begin the abstraction, the abstractor chose the subject ID from a pull down list on the Demographic, Breast Cancer and Treatment Form and all electronic data available for this ID would fill the corresponding data fields in the electronic abstraction forms. Each abstraction began with verification of the eligibility criteria, allowing in one step the abstractor to continue if further record abstraction was indicated or to stop immediately and move on to the next case. Upon confirmation of eligibility, stage, age and race, if the sampling quota had been met for the specified group, the abstractor was prompted to move on to the next subject. The EDC operated on the premise that all cases were in the process of completion, thereby allowing editing of all pre-filled data elements. Extensive quality control procedures were included in the EDC to minimize abstraction error. These procedures included (1) range checks for dates and value responses, (2) logic checks prompting the abstractor to verify the answer if it was either an out-of-range value or the response was not feasible given other clinical information, and (3) command buttons with embedded coding information to assist in data abstraction. The abstractor was able to set the abstraction to complete electronically only when all data and logic checks had been satisfied. Once the data abstraction was complete and passed the pre-programmed check for completeness and logical consistency (Final Check), the record was exported to the "back-end" database. At the beginning of each month, all data were sent via a secure internet transfer site to the data coordinating site at Boston Medical Center in electronic format. Data from each electronic source within and across participating sites were merged and cleaned at the data coordinating site in preparation for analyses, using SAS statistical software.[10]

### The inter-/intra-rater reliability (IRR) system

We used a completely automated triple abstraction process to incorporate quality controls to reduce inter- and intra-site variability and to improve accuracy of data collection. We developed a Microsoft^® ^Access-based IRR system using a modified version of the automated EDC system. The IRR system contained a subset of 54 key data elements (of 658 collected by the EDC), and was organized on a single form with tabular format to differentiate five areas of interest for evaluating data reliability and consistency: tumor characteristics, tumor treatment, development of recurrence or second primary, comorbidity, and surveillance mammography. The same range checks, logic checks, and a final check for completion as in the EDC system were programmed for these key variables. The IRR system contained in its "back end" tables the data from original abstractions on five records for each abstractor, randomly selected from records completed three months prior to the IRR exercise. For re-abstractions, record for each subject ID in the IRR system was pre-populated with the same data that was preloaded into the EDC system for the original medical record abstraction.

The key data elements were re-abstracted and entered directly into the IRR system by the original abstractor (for intra-rater reliability comparison) and by the site project manager (for inter-rater reliability comparison). Upon completion of all five records by an abstractor, the IRR system compared re-abstracted data with the original abstraction and the pre-programmed reporting function in the IRR system provided both the number of mismatches and percent agreement for each record re-abstracted and by sub-areas in the abstraction instrument. The IRR data were sent by each site to the Boston Medical Center and pooled to determine a study-wide reliability rate. Reports were generated for each abstractor by subject ID, comparing the re-abstractions to the original abstraction, aggregated by content sections of compared data (tumor characteristics, treatment, recurrence/second primary, comorbidity, and surveillance mammography), and disaggregated into each data element. The number of mismatches and percent agreement were electronically calculated for all items by subject ID and automatically displayed in three categories: total, preloaded variables, and non-preloaded variables. Data were shared with the abstractors to develop strategies to reduce errors. IRR exercises were conducted once each during the first and second halves of the data collection period.

## Results

The EDC system and a user manual with detailed coding guide were developed concurrently over a 6-month period at Boston Medical Center, the study data-coordinating center. Both the EDC and the manual were tested at all sites during the training sessions and the revised versions were released one month later. Each site had 1 to 3 abstractors working on the study as well as a part time resident programmer who was knowledgeable in Microsoft^® ^Access and SAS. Depending on where the medical records were stored, data abstraction took place at multiple locations, up to 20 at one CRN site. Fifteen medical record abstractors were trained to use the EDC system and subsequently evaluated with the IRR system.

There were varying amounts of electronic data available from cancer registry and administrative databases for preloading. Two non-registry sites had 5 data items available (age, birth-month, birth-year, potential diagnosis date, end of follow-up date), whereas four registry sites had 32 to 37 data items available. All electronically available data were sent in Microsoft^® ^Excel, Access, or SAS format and merged to the "back end" database of the EDC system.

Of 3,766 potential cases identified at 6 sites, electronic data on 3,124 cases were preloaded into the EDC. The remaining cases not included in the EDC were from Kaiser Permanente Southern California where only 10% of the younger than 80 years age, non-Hispanic white, stage I cases were sampled. A sampling fraction of 50% was implemented initially at the other 5 sites for the same subgroup, however, based on sociodemographic and disease characteristics necessary to meet our scientific goals, we decided to enroll all identified cases at these sites. We were able to implement and adjust sampling strategies by modification of the preloaded case lists. This real time adjustment of sampling characteristics allowed us to compensate during the data collection process.

Eligibility was confirmed for 1,859 cases and data collection was completed 18 months after release of EDC. There were 49 cases (of 3,124 total preloaded) electronically identified during the data collection process as missed abstractions; 15 of those were found to be enrollment-eligible and were subsquently abstracted. This process also allowed us to capture data on why the abstraction was not done, thus providing information on potential selection biases by site that otherwise would not have been available. For example, we were able to examine the preloaded characteristics of the 257 women with missing medical records to determine whether this subgroup was different from the 1,859 women who were included in the study cohort.

**Figure 2:**
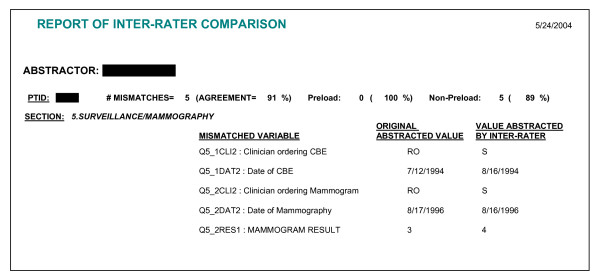
Example of inter-rater reliability comparison report.

We developed the IRR system directly from the EDC system within a 2-month period. Programming time necessary for development of the IRR system was reduced substantially by the fact that all data structures in the IRR system were derived from the original EDC system. Additionally, the menu-driven format of the IRR system, which was based on the EDC system, was familiar to the abstractors and therefore easy and quick to implement with no further training. Figure 2 displays an example of an IRR report. All discrepancies in the reports were adjudicated at the sites. We were able to identify from the IRR summary table, as shown in Table 1, the possible inconsistency in the abstraction of last known date of hormonal therapy. This inconsistency prompted us to verify our data against electronically available pharmacy data; the agreement was over 83%.

**Table 1:** Summary of inter-rater reliability exercise for first half of data collection period, by type of data elements.

Site/Abstractor	Tumor	Treatment	Treatment* w/o HT Date	Recurrence	Comorbidity	Surveillance
A/1	3	6	2	3	2	12
A/2	1	6	3	1	3	19
						
B/3	3	6	2	7	0	11
B/4	1	9	4	6	4	10
B/5	0	5	1	1	0	15
						
C/6	0	5	2	0	1	27
C/7	0	3	2	1	5	13
						
D/8	2	5	3	1	3	15
D/10	5	7	6	0	0	5
D/11	1	7	3	2	1	11
						
E/12	2	7	5	1	3	8
						
F/15	8	7	2	0	1	7

#Disagreements	26	73	35	23	23	153
Total in section	420	360	360	360	1140	960
%Disagreement	6%	20%	10%	6%	2%	16%

*Excluding 38 Disagreements on Last Known Date of Hormonal Therapy

Data cleaning after the completion of data collection consisted of running a SAS program to identify inconsistencies and coordinating with sites to resolve those inconsistencies in four main areas: (1) side of the breast (same vs. opposite breast from primary tumor) for recurrence and second primary cases; (2) follow-up time based on the reason for end of follow-up (death, disenrollment or completion of the 10 year study follow-up period); (3) hormonal therapy start and stop dates; and (4) completeness of surgeon data.

**Figure 3:**
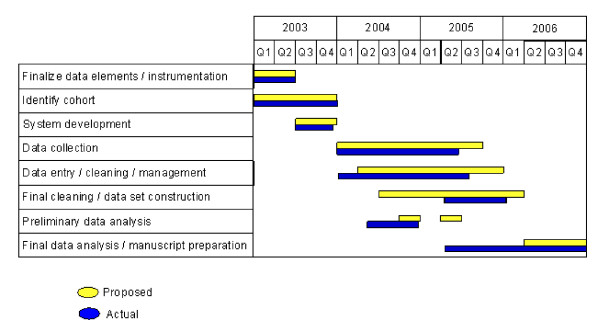
Actual versus proposed study timeline.

These efficiencies resulted in a substantial reduction in the time needed for data management. The cumulative effect was almost four months in reduced data collection time compared to the original project schedule and almost 12 months reduction in time to manuscript preparation (Figure 3). Using the third project year salaries and time allocations, we calculated the dollar savings at each of our six data collection sites as the sum of two components: (1) 25% of budgeted project manager time for four months, and (2) budgeted medical record abstractor time for four months. With an additional 50% savings of budgeted data analyst time for four months at the Boston Medical Center, a savings of $72,000 in total from reduced data collection costs across the six sites was estimated.

## Discussion

Our experience with EDC resulted in significant research advantages. The resource efficiency of the study was derived directly from the development of our EDC system, which combined medical record abstraction and data entry into one step so that real-time data cleaning was possible, and once the abstraction was complete, data were immediately available for analysis. EDC allowed for ease and efficiencies in data management between sites and greatly increased overall study data flow. One benefit was the ablity to monitor site-specific progress, sample characterics, and cases deferred for later abstraction when follow-up had not been completed. The component of the EDC – which allowed deferred cases to be identified before data abstraction began – eliminated the need to order medical records twice. A second benefit was the ability to track missed abstractions. Before the start of data collection, all of our study sites identified all potentially eligible breast cancer cases through the use of electronic databases. We loaded these lists of pre-identifed subjects into the EDC system before data collection. As a result, the total number of possible cases for each site was pre-determined and we were able to identify in a timely manner and with ease the cases that were missed during the medical record abstraction process, a step that would not have been possible with full paper data collection.

With increasing availability of electronic data for research and quality measurement, there is an increasing need for ascertaining the accuracy of the data. This was reported by Haque, et al. in relation to colorectal cancer screening.[11] We found that various components of the EDC such as the eligibility verification algorithm, the sampling algorithm, the subject deferment algorithm and the final logic check algorithm contributed to improve data collection efficiency. In addition, Microsoft^® ^Access-based programming allowed data quality checks at various levels so that the final data-cleaning step was minimized.

The most important innovation and efficiency associated with our use of EDC was our ability to automate the IRR process. We developed inter- and intra- reliability modules that allowed for interim feedback during data collection. This interim feedback cycle allowed real-time corrective action during the data collection process based on the IRR findings. Comparison of triple-abstractions using electronic forms can be done rapidly, but requires writing and testing programs within the structure of the EDC database. We simplified identification of errors and disagreements among abstractors and across sites by the automated IRR process.

We have summarized in Table 2 the strengths of EDC and highlighted items for consideration when deciding on use of EDC versus paper data collection process. Length and complexity of the data collection instrument, availability of data (electronic versus paper medical record), and complexity of the abstraction process (number of abstraction locations, number of abstractors) are key elements to consider during the decision making process. In addition, costs associated with equipment, system development and training need to be evaluated. When using EDC, not only can overall record review cost be substantially reduced, but it can also be carried out more efficiently. Development, interim support, and troubleshooting are simply the marginal costs, which can be offset by gains from an EDC system.

**Table 2:** Strengths and considerations of an electronic data collection system.

Strengths	Considerations
Flexible with amount and type of site-specific electronic data available for preloading	May not be cost-effective if the data collection instrument is short, there is no mixed data type and/or number of data abstraction sites is small
Combines data abstraction and data entry into one step and allows for real-time data cleaning, so that data were immediately available for analysis	Resources needed for 1. system and manual development 2. training 3. hardware 4. software
Ease of managing data from multiple locations	Skilled personnel needed at each site throughout the study period
Efficiency in data management from 1. automated algorithm for eligibility verification 2. integrated sampling scheme so that abstraction can be stopped once the sampling quota is reached 3. algorithm to defer abstraction until follow up period is complete	
Readily allows for development of subsequent systems such as automated Inter-rater reliability process	

Any use of automated EDC for research purposes should follow these guidelines. First, the electronic data collection form should be programmed only after designing, piloting, and refining a paper version (pilot testing should involve more than one abstractor and/or site when conducting a multi-site study). This is a logical argument based on the fact that multiple revisions to a data collection instrument are expected in a large scale research study, especially when multiple sites are involved. Revisions made on paper are cheaper than the programmer time required for revisions to the EDC. Second, the data collection form should be designed with the needs of the data entry personnel in mind (*e*.*g*., structure the form in a logical manner and make coding of responses as consistent and straightforward as possible). Usability issues related to EDC for clinical trials have been discussed by Schmier, et. al., where it has been reported to contribute to decreased costs, enhanced quality of the data, and minimized time to analysis.[12] Finally, a real time quality control mechanism needs to be in place for assessing and correcting data entry errors. The centralized generation of monthly data-gathering provided early and ongoing indications of site-specific and generalized data collection problems, thus providing opportunity for real time corrective actions, such as obtaining an additional abstractor at one site after observing a slower than expected rate of abstract completion.

## Conclusion

There is no more important aspect of research than to acquire high quality data, which is highly reliant on the quality of the data collection process. It follows, therefore, that the design, development and quality assurance of EDC should be given meticulous attention. Automated EDC provides an efficient and innovative platform well suited to allow researchers to capture, manage and report on study progress and data quality. The flexibility of EDC allows for multi-site as well as longitudinal studies. EDC permits the seamless management of multiple workflows and protocol algorithms, and the efficient deployment and management of studies, which can be a critical success factor in any research.

## Competing interests

The author(s) declare that they have no competing interests.

## Authors' contributions

With theoretical and editorial feedback from TLL and RAS, SST developed the programming and KMCG developed the documentation for the EDC and IRR systems. MCM trained the users at all sites. SHA implemented the systems at Henry Ford Health System, DSMB implemented the systems at Group Health, SME implemented the systems at Kaiser Permanente Southern California, TSF implemented the systems at Fallon Community Health Plan, FF implemented the systems at Lovelace Health Plan, and FFW implemented the systems at Health Partners. This manuscript was drafted by SST and KMCG, but all authors read, provided feedback on earlier drafts of the manuscript, and approved the final manuscript.

## Pre-publication history

The pre-publication history for this paper can be accessed here:


